# Prediabetes Associates with Matrix Metalloproteinase-8 Activation and Contributes to the Rapid Destruction of Periodontal Tissues

**DOI:** 10.1055/s-0044-1788797

**Published:** 2024-10-01

**Authors:** Kehinde Adesola Umeizudike, Nur Rahman Ahmad Seno Aji, Katariina Niskanen, Iina Rantala, Dimitra Sakellari, Andreas Grigoriadis, Tommi Pätilä, Shipra Gupta, Timo Sorsa, Ismo T. Räisänen

**Affiliations:** 1 Department of Oral and Maxillofacial Diseases, Head and Neck Center, University of Helsinki and Helsinki University Hospital, Helsinki, Finland; 2 Department of Preventive Dentistry, Faculty of Dental Sciences, College of Medicine, University of Lagos, Lagos, Nigeria; 3 Department of Periodontics, Faculty of Dentistry, Universitas Gadjah Mada, Yogyakarta, Indonesia; 4 Department of Preventive Dentistry, Periodontology and Implant Biology, Dental School, Aristotle University of Thessaloniki, Thessaloniki, Greece; 5 Department of Preventive Dentistry, Periodontology and Implant Biology, Dental School, Aristotle University of Thessaloniki, Thessaloniki, Greece; 6 Department of Pediatric Surgery, Children's Hospital University, University of Helsinki and Helsinki University Hospital, Helsinki, Finland; 7 Unit of Periodontology, Oral Health Sciences Centre, Post Graduate Institute of Medical Education and Research, Chandigarh, India; 8 Division of Periodontology, Department of Dental Medicine, Karolinska Institutet, Stockholm, Sweden

**Keywords:** periodontitis, diagnosis, matrix metalloproteinase 8, biomarkers, point-of-care testing, prediabetes, diabetes

## Abstract

**Objective** The aim of this cross-sectional study was to investigate the relationship between periodontitis, potential periodontitis oral fluid biomarkers, and prediabetes.

**Materials and Methods** This study included 150 Greek adults aged 25 to 78 years who were tested with an Hemoglobin A1C (HBA1c) diagnostic system, an active-matrix metalloproteinase-8 (aMMP-8) point-of-care (PoC) test, and several salivary biomarkers enzyme-linked immunosorbent assay tests and gelatin zymography. A full-mouth clinical examination was performed to assess their periodontal and oral health status.

**Statistical Analysis** The Kruskal–Wallis test was used to determine the statistically significant difference in the levels of periodontal oral fluid biomarkers between the different periodontitis stages, periodontitis grades, and the stages and grades of periodontitis combined. Spearman's rank correlation was performed to assess the strength and direction of the association between aMMP-8 and HbA1c levels (<5.7 and ≥5.7%) and with the other oral fluid biomarkers among patients with severe periodontitis. A two-sided *p* -value below 0.05 was considered statistically significant in this study.

**Results** aMMP-8, but not total MMP-8 or other biomarkers, associated significantly with the stage and grade of periodontitis combined ( *p* < 0.001, Kruskal–Wallis test). Among stage III grade C periodontitis patients, aMMP-8 levels were significantly positively correlated with prediabetes (Spearman's rho = 0.646, *p* = 0.044), total MMP-8 (rho = 0.636, *p* = 0.048), PMN Elastase (rho = 0.729, *p* = 0.017), total MMP-9 (rho = 0.721, *p* = 0.019), and total MMP-8/TIMP-1 molar ratio (rho = 0.879, *p* < 0.001).

**Conclusion** Prediabetic disease development can upregulate MMP-8 expression (total MMP-8) in rapidly progressing, severe periodontitis, where MMP-8 latent species are further activated into their active forms (aMMP-8). Simultaneously, several proinflammatory biomarker levels are elevated in this tissue-destructive biomarker cascade. This development is easily detectable online/in real-time within 5 minutes by aMMP-8 PoC testing at the dentist's office.

## Introduction

The bidirectional relationship between the chronic noncommunicable diseases periodontitis and diabetes has been well-established for a long time. 1 2 Diabetes is a major risk factor for periodontitis and (uncontrolled) diabetes with elevated HbA1c levels (hyperglycemia), predisposes individuals to more severe and rapid progression of periodontitis. 3 4 Oral fluid biomarkers for periodontitis have been studied extensively. 5 6 7 These include, among others, matrix metalloproteinase (MMP) 8 (MMP-8) and MMP-13 and gelatinases, MMP-2 and MMP-9. 6 7 Compelling evidence support a more direct role for active-matrix metalloproteinase-8 (aMMP-8) in the progressive loss of periodontal connective tissues, compared with latent or total MMP-8. 6 8 9 10 11 12 13 14 15 It is necessary to distinguish between aMMP-8 and latent or total MMP-8 in oral health biomarker studies, as the latent MMP-8 is enzymatically inactive and requires activation by other host and microbial proteases to become aMMP-8, which has the collagenolytic and proteolytic activity. 10 16 Thus, aMMP-8 testing can be utilized to monitor the levels of tissue destructive aMMP-8 in oral fluids and assess periodontitis progression and periodontal treatment effect. 9 16 17 18 19 20 21 22 23

Previously, a positive association was reported between aMMP-8 and the staging and grading of periodontitis, 9 according to the 2018 classification of periodontitis. 24 Furthermore, aMMP-8 levels in mouthrinse are significantly lower in healthy patients in comparison to patients with more advanced levels of periodontitis. Additionally, a potential aMMP-8–periodontitis–prediabetes/diabetes connection has been suggested earlier, implying a positive association between aMMP-8, and both periodontopathogenic dysbiotic proteolysis and prediabetes, as well as with periodontitis grade. 25 There is also an increasing trend in MMP-8 protein expression levels in patients suffering from either periodontal disease and diabetes or both. 26 Diabetes/hyperglycemia increases the local periodontal inflammation and related inflammatory biomarker levels in periodontal tissues. 27 These heightened inflammatory responses through the increased secretion of cytokines like IL-1β, TNF-α, and IL-6, oxidative stress, disrupt the Receptor activator of nuclear factor kappa-b ligand (RANKL)/Osteoprotegerin (OPG) axis, thereby promoting rapid breakdown of the periodontal connective tissues, alveolar bone resorption, exacerbating periodontitis. 27

The aim of this study was to further investigate and gain a deeper understanding of the relationship between periodontitis, potential oral fluid biomarkers including mouthrinse aMMP-8, aMMP-9, tMMP-8, tMMP-9, PMN elastase, MPO, TIMP-1, IL-6, Calprotectin, and prediabetes. We hypothesized that aMMP-8 levels in patients with periodontitis are associated with hyperglycemia that can amplify the inflammation in periodontal tissues and eventually the progression of tissue destruction. Furthermore, aMMP-8 levels in periodontitis patients are associated with various other periodontal tissue destructive enzymes linked to a complex and diverse pathologic periodontitis development.

## Materials and Methods

One hundred and fifty Greek adult patients aged 25 to 78 years, attending the undergraduate and postgraduate clinics of the Department of Periodontology (Dental School of the Aristotle University, Thessaloniki, Greece) and the Periodontal Department of 424 General Army Hospital (Thessaloniki, Greece) were recruited to this study. They were selected from the sample pool of 731 potential patients, at high risk of developing diabetes mellitus, determined as a score > 9 in the self-assessed questionnaire proposed by the Centers for Disease Control and Prevention (CDC, Atlanta, GA) Prediabetes Screening Test (National Diabetes Prevention Program) described by Grigoriadis et al. 28 The minimum sample size of 139 adults to identify undiagnosed diabetes was calculated by using the mean percentage of undiagnosed diabetes in Europe (10%) together with the statistical equation for population frequency as previously described. 28

All patients underwent full periodontal examination performed by one calibrated examiner (A.G). Periodontal stage and grade were defined based on the information collected by periodontal examination and radiographic bone loss on orthopantomographic digital images according to the 2018 classification system of periodontal diseases. 2 24 Exclusion criteria were as follows: age under 18 years, patient's physical status ≥3 in the American Society of Anesthesiologists classification, presence of diabetes mellitus or immunomodulatory diseases, medical diagnosis of rheumatoid arthritis by a rheumatologist, medication intake affecting glycemic control, periodontal therapy within the last 6 months, presence of less than 20 teeth, and pregnant or lactating women. The study was approved by the Ethical Committee of the School of Dentistry (protocol number #64, 06/12/2018) and conducted according to the protocol outlined by the Research Committee, Aristotle University of Thessaloniki, Greece. Participants signed an informed consent. All procedures performed in the present study involving human participants were in accordance with the ethical standards of the institutional and/or national research committee and complied with the Declaration of Helsinki.

### Oral Fluid Sample Collections, HbA1c, and Biomarker Analysis Protocols

The participants fulfilling the CDC criteria for developing type 2 diabetes were tested with the Cobas® b101 (Roche Diagnostics, Hoffmann La Roche, Mannheim, Germany) to measure their HbA1c levels in human capillary blood (from a patient's finger) by photometric transmission procedure as described in Grigoriadis et al. 28 The method has been standardized against the International Federation of Clinical Chemists reference method. 29 All subjects in this study identified with hyperglycemia (HbA1c ≥ 5.7%) were strongly advised to contact their physician for further consultation, and laboratory tests to decrease their risk of developing type 2 diabetes.

Patients were provided mouthrinse samples for quantitative aMMP-8 point-of-care (PoC) testing. They were instructed to refrain from eating or brushing their teeth 1 hour before sampling. The procedure of the mouthrinse began with the patients rinsing their mouth with drinking water for 30 seconds and then spitting the water out. After a 60-second wait, they rinsed again with 5 mL of distilled water for 30 seconds and then expectorated into a measuring cup. Following the manufacturer's instructions, the sample was drawn from the measuring cup with a syringe, filtered, and transferred to the aMMP-8 Periosafe® kit test cassette. The quantitative aMMP-8 result was analyzed within 5 minutes by the commercially available digital reader ORALyzer® (Dentognostics GmbH, Jena, Germany). Additionally, aMMP-8/NTP (per number of teeth present) was calculated as described previously by Deng et al. 21

Stimulated salivary samples (∼5 mL) were also collected before the clinical examination. Before saliva collection, patients rinsed their mouths with water and spat the water out. Then they chewed a small piece of paraffin wax, to facilitate saliva secretion, which was swallowed for the first 30 seconds, after which the excreted saliva was collected for the next 5 minutes into a plastic tube. The stimulated saliva samples were immediately centrifuged at 1,000 × g for 5 minutes before being frozen at −20 °C and stored at −80 °C until enzyme analysis was performed.

Commercially available enzyme-linked immunosorbent assay (ELISA) kits were used to analyze saliva samples for total MMP-8 (R&D Systems, Minneapolis, MN), total MMP-9 (R&D Systems, Minneapolis, MN), Human PMN Elastase (Bender MedSystems GmbH, Wien, Austria), MPO (Immundiagnostik AG, Bensheim, Germany), TIMP-1 (Human Biotrak ELISA systems, Amersham Biosciences, GE Healthcare, Buckinghamshire, United Kingdom), IL-6 (R&D Systems, Minneapolis, MN), Calprotectin (R&D Systems, Minneapolis, MN) and gelatin zymography analysis for active MMP-9 (aMMP-9) according to the instructions of the manufacturers. 28 30

The protocols for the ELISA kits were summarized as follows: Protein levels were measured using precoated wells by adding samples and standards at room temperature. The secondary antibody in each kit was conjugated with horseradish peroxidase. Tetramethylbenzidine was used as a substrate. The concentration of the analyzed proteins was read by optical density, while the absorbance was measured using a wavelength of 450 nm using a Victor X4 Multilabel Reader (PerkinElmer Finland Oy, Turku, Finland). Based on measured standards, the calibration curve was drawn and used to calculate the concentration of the measured proteins. The detection limits were agreed upon based on each kit used.

Gelatin zymography analysis was used to analyze gelatinolytic activity in the saliva samples for aMMP-9 using 11% SDS-polyacrylamide gels impregnated with 1 mg/mL gelatin (Merck) as substrate. 30 Before the electrophoresis, the samples were mixed at room temperature with Laemmli's sample buffer for a 2-hour incubation time and without any reagent reduction. Prestained low-range molecular weight SDS-PAGE standards (Bio-Rad) were used as molecular weight markers and human MMP-9 (Merck) as a positive control. After electrophoresis, the gels were washed two times with 50 mM Tris-HCl buffer and then incubated in 50 mM Tris-HCl buffer solutions that had pH 7.5 and contained 0.02% of NaN _3_ , 0.5 mM CaCl _2_ , and 1 μM ZnCl _2_ overnight at 37 °C. The gelatinolytic activity was then visualized with 1% Coomassie Brilliant Blue R 250 solution and detected as clear bands on the stained gel. The intensities of gelatinolytic activities were quantified with the GS-700 Imaging Densitometer Scanner using Quantity One-program (Bio-Rad). Finally, the activity of aMMP-9 was calculated as a percentage from the optical densitometer values of pro- and aMMP-9 forms and proMMP-9, respectively.

### Statistical Methods

The normality of the measured variables was inspected with the Shapiro–Wilk test and visually. Kruskal–Wallis test was used to determine if there was a statistically significant difference in the levels of periodontal oral fluid biomarkers between the different periodontitis stages, periodontitis grades, and the stages and grades of periodontitis combined. Furthermore, pairwise comparisons were performed by the Dunn–Bonferroni test. Finally, Spearman's rank correlation was performed to assess the strength and direction of the association between aMMP-8 and HbA1c levels (<5.7 and ≥5.7%) and with the other oral fluid biomarkers among patients with severe periodontitis. A two-sided *p* -value below 0.05 was considered statistically significant in this study. Statistical analyses were performed with the SPSS Base 29.0.0.0 Statistical Software Package (SPSS Inc., IBM, Chicago, IL).

## Results

**Fig. 1:**
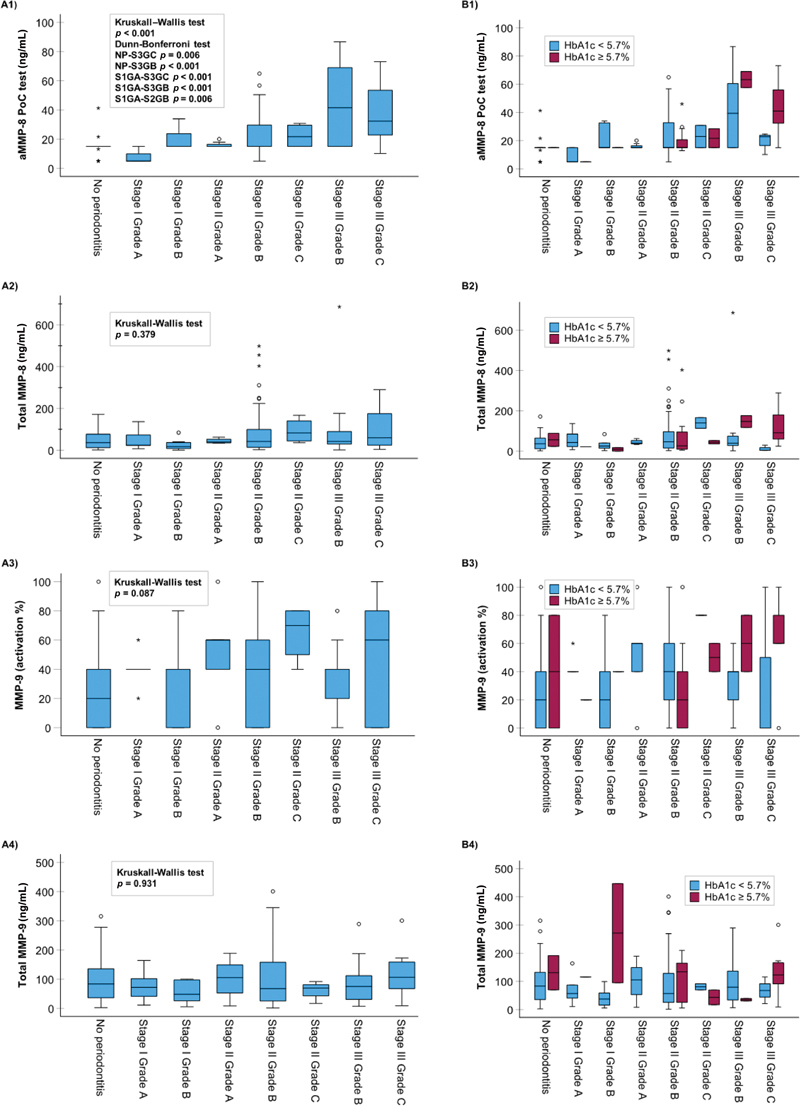
The relationship between stage and grade of periodontitis combined and (1) aMMP-8 (ng/mL), (2) total MMP-8 (ng/mL), (3) aMMP-9 (%), and (4) total MMP-9 (ng/mL). Panel ( **A** ) without categorization on the basis of HbA1c levels and panel ( **B** ) following categorization on the basis of HbA1c levels ( *N* = 150 Greek adults). aMMP-8, active-matrix metalloproteinase-8; MMP, matrix metalloproteinase.

Table 1 presents patient characteristics of the patient population. Supplementary Table S1 (available in the online version only) and Figures 1 2 3 present the association of the stage of periodontitis, grade of periodontitis, and their combination, with the oral fluid biomarkers tested in this study. The only biomarker found to have a statistically significant association with the three different periodontitis classifications was aMMP-8 measured by the PoC test ( *p* < 0.001) ( Table 1 and Fig. 1 ). Furthermore, aMMP-8 PoC test /NTP was significantly associated with the stage and grade of periodontitis as well ( *p* < 0.001; Table 1 ). The other oral fluid biomarkers had no significant associations with the stage or grade of periodontitis, or if they did, they associated with only one of them without any significant pairwise differences and not with the stage and grade of periodontitis combined ( Table 1 and Figs. 1 2 3 ). This extends the previously published significant associations between the aMMP-8 PoC test (with and without NTP) and stage and grade of periodontitis, and further affirms the evidence that there is no significant relationship between total MMP-8 and periodontitis stage in the same population of Greek adults. 9 10 25

**Table 1:** Patient characteristics according to periodontal condition

	No periodontitis	Stage I Grade A	Stage I Grade B	Stage II Grade A	Stage II Grade B	Stage II Grade C	Stage III Grade B	Stage III Grade C	*p* -Value
Age (years); mean ± SD	43.3 ± 12.8	61.1 ± 9.2	62.1 ± 7.6	60.1 ± 13.6	54.2 ± 9.3	60.0 ± 9.8	55.9 ± 10.6	56.1 ± 8.7	0.003
Gender (women/men)	11	7	7	5	31	3	9	3	0.011
	20	0	1	2	39	1	4	7	
Smoking (yes/no)	3	3	5	0	22	2	5	5	0.026
	25	4	3	7	46	2	8	5	
Electronic smoking	3	0	0	0	2	0	0	0	–
Parent with diabetes (yes/no)	22	6	3	5	40	3	10	7	0.396
	9	1	5	2	30	1	3	3	
Body mass index (kg/m ^2^ )	30.6 ± 4.5	29.2 ± 5.8	27.8 ± 2.6	28.1 ± 2.7	30.5 ± 4.6	32.8 ± 8.5	28.8 ± 6.6	30.1 ± 5.6	0.312
HbA1c (%)	5.21 ± 0.35	5.26 ± 0.59	5.44 ± 0.36	5.24 ± 0.29	5.29 ± 0.60	6.25 ± 1.32	5.35 ± 0.32	6.29 ± 1.15	0.185
Frequencies of HbA1c <5.7%	29	6	6	7	55	2	11	3	
HbA1c = 5.7–6.5%	2	1	2	0	13	1	2	3	
HbA1c = 6.8%	0	0	0	0	1	0	0	2	
HbA1c = 6.9%	0	0	0	0	0	0	0	1	
HbA1c = 8.1%	0	0	0	0	1	0	0	0	
HbA1c = 8.2%	0	0	0	0	0	1	0	0	
HbA1c = 8.9%	0	0	0	0	0	0	0	1	
Extent of periodontitis (localized/generalized)	–	2	2	0	3	0	0	0	0.049
		5	6	7	67	4	13	10	
Number of teeth	26.8 ± 1.9	24.6 ± 2.8	24.0 ± 2.4	23.1 ± 2.7	24.6 ± 2.9	23.5 ± 3.0	22.7 ± 3.4	21.6 ± 4.5	<0.001
Periodontal probing depth (mm); mean ± SD	2.3 ± 0.3	2.1 ± 0.4	2.3 ± 0.5	2.7 ± 0.2	3.0 ± 0.7	3.3 ± 1.1	3.7 ± 0.8	4.1 ± 1.0	<0.001
Bleeding on probing (%)	42.2 ± 25.5	23.6 ± 9.8	49.0 ± 27.1	48.1 ± 15.8	55.2 ± 23.0	76.6 ± 10.3	55.7 ± 19.1	72.8 ± 23.7	<0.001
Visible plaque index (%)	43.5 ± 22.2	32.0 ± 16.0	36.1 ± 34.5	45.0 ± 28.4	46.2 ± 26.7	81.1 ± 16.9	53.5 ± 31.7	76.0 ± 16.1	<0.001

Abbreviation: SD, standard deviation.

*p* -Values for variables gender, smoking, parent with diabetes and extent of periodontitis were calculated by Pearson's chi-square test (exact and two-sided) periodontal condition. *p-* Values for age, body mass index, number of teeth, bleeding on probing, visible plaque index and periodontal probing depth were calculated by Welch's *t* -test (two-sided) for periodontal condition.

**Fig. 2:**
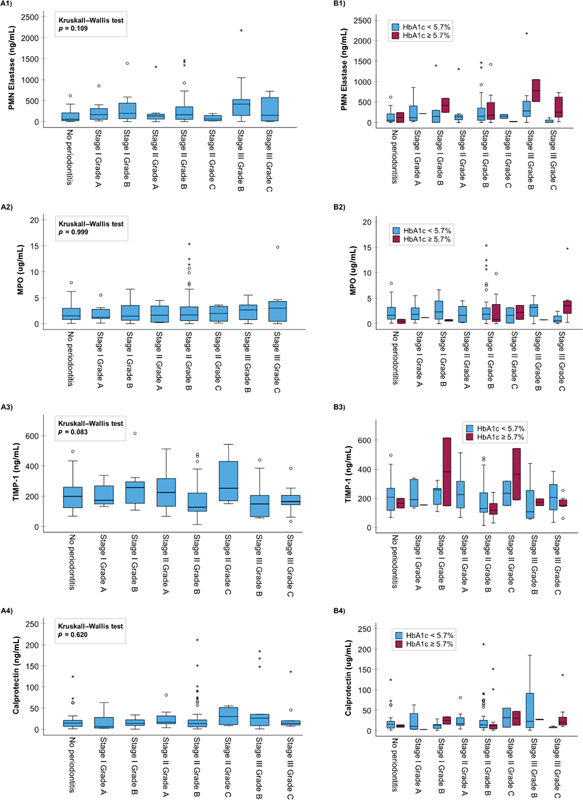
The relationship between stage and grade of periodontitis combined and (1) PMN Elastase (ng/mL), (2) MPO (ng/mL), (3) TIMP-1 (ng/mL), and (4) Calprotectin (ng/mL). Panel ( **A** ) without categorization on the basis of HbA1c levels and panel ( **B** ) following categorization on the basis of HbA1c levels ( *N* = 150 Greek adults).

**Fig. 3:**
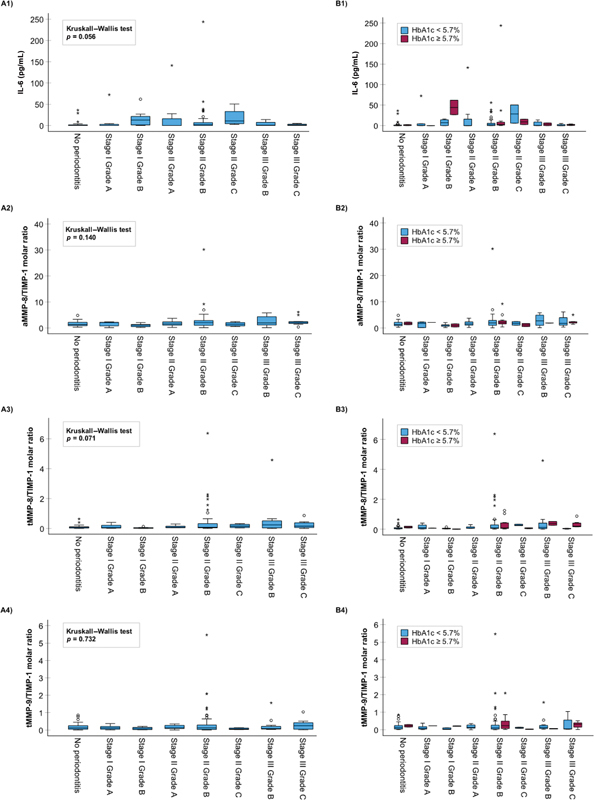
The relationship between stage and grade of periodontitis combined and (1) IL-6 (ng/mL), (2) active MMP-8/TIMP-1 molar ratio, (3) total MMP-8/TIMP-1 molar ratio, and (4) total MMP-9/TIMP-1 molar ratio. Panel ( **A** ) without categorization on the basis of HbA1c levels and panel ( **B** ) following categorization on the basis of HbA1c levels ( *N* = 150 Greek adults). MMP, matrix metalloproteinase.

Figures 1^2^,3 present the relationship between the oral fluid biomarkers and periodontitis category based on HbA1c (<5.7 and ≥5.7%). As shown in Fig. 1 , the stage and grade of periodontitis were associated positively with aMMP-8 (also categorized by the HbA1c), while there was no relationship between the stage and grade of periodontitis and total MMP-8. Finally, among stage III grade C periodontitis patients, aMMP-8 levels were significantly positively correlated with HbA1c levels (Spearman's rho = 0.646, *p* = 0.044), as well as, with total MMP-8 (rho = 0.636, *p* = 0.048), PMN Elastase (rho = 0.729, *p* = 0.017), total MMP-9 (rho = 0.721, *p* = 0.019) and total MMP-8/TIMP-1 molar ratio (rho = 0.879, *p* < 0.001). On the whole, in stage III patients, aMMP-8 correlated with total MMP-8 (rho = 0.642, *p* < 0.001), PMN Elastase (rho = 0.655, *p* < 0.001), active MMP-8/TIMP-1 molar ratio (rho = 0.458, *p* = 0.028), and total MMP-8/TIMP-1 molar ratio (rho = 0.719, *p* < 0.001).

## Discussion

The critical discovery in this study was the effectiveness of aMMP-8 oral fluid chairside/PoC testing as a diagnostic tool for screening and predicting the risk for both periodontitis and prediabetes compared with several other biomarkers. Our first hypothesis about the association between aMMP-8 and hyperglycemia (HbA1c) in patients with periodontitis was supported by the results of this study. As the stage and grade of periodontitis increased, higher aMMP-8 levels were observed in patients with elevated HbA1c levels. This relationship between aMMP-8 and hyperglycemia (HbA1c) is in agreement with the current literature. There is compelling evidence supporting that aMMP-8 but not total or latent MMP-8 plays a direct role in the progressive destruction of the periodontal connective tissue. 8 10 11 31 On the other hand, hyperglycemia is associated with the progression of periodontitis. 32 Additionally, the study lends support to the second hypothesis of this study, that the activation of a cascade of oxidative and proteolytic enzymes associated with tissue destruction is linked to this pathologic disease development. Our data indicate that the development of diabetes can increase the expression of MMP-8 (total MMP-8) in severe periodontitis cases. Recently, Miller et al (2023) observed elevated total MMP-8 levels in patients with Type 2 Diabetes Mellitus (T2DM) and periodontitis. 33 However, as demonstrated by the current study, most of the total MMP-8 is then in active form as opposed to the latent/pro-form MMP-8. The activation of MMP-8 indicates that high levels of HbA1c are a contributing factor to the progression of periodontitis, particularly in the advanced stages of the disease. 34 35 This activation may also indicate the development of diabetes, which is known to upregulate and trigger collagenolytic MMP-8 in the gingival tissues and oral fluids. 26 36 Elevated MMP-8 activation has also been recorded systemically in diabetes, diabetic cutaneous wound fluids, and diabetic nephropathy. 37 38 MMP-8 can degrade insulin receptors, decreasing insulin sensitivity and increasing susceptibility to prediabetes, which can be prevented by using a synthetic MMP-8 inhibitor such as doxycycline. 38 39

The bidirectional relationship between diabetes and periodontitis is widely recognized in academic literature. 1 27 40 While individuals with untreated or poorly controlled diabetes are at a two to three times higher risk for developing periodontitis, periodontitis increases HbA1c levels and diabetic complications in people living with T2DM. 27 In individuals without diabetes, severe periodontitis is associated with higher HbA1c levels and prevalence of prediabetes compared with periodontally healthy individuals. 41 The current study observed that patients with severe periodontitis who developed (pre)diabetes and had elevated HbA1c levels were more prone to having a cascade of tissue-destructive enzymes than patients with normal HbA1c levels. aMMP-8 is a major part of the periodontal tissue destructive process and reflects and predicts active, progressive periodontal degeneration. 6 8 The present study discovered a connection between aMMP-8 and several oxidative and proteolytic tissue destructive enzymes in severe periodontitis with (pre)diabetes disease progression. Thus, elevated levels of aMMP-8 may predict not only the presence, initiation, and development of periodontitis but also diabetes disease progression. Additionally, the present results reveal that aMMP-8 oral fluid levels can simultaneously serve as an indicator for the increased activity of the tissue-destructive enzyme cascade in periodontitis cases.

Periodontitis and its ensuing progression of tissue destruction are associated with elevated aMMP-8 levels. 11 31 As the present study suggests, the development of prediabetes can increase total MMP-8 levels in severe periodontitis cases. Subsequently, periodontal disease induces the conversion of the inactive latent/pro-forms of total MMP-8 into its active collagenolytic form (aMMP-8), which can be easily detected using aMMP-8 PoC test kits. 9 17 18 19 21 25 Consequently, patients with both severe periodontitis and blood sugar imbalances have abnormally elevated levels of aMMP-8 in their mouthrinse samples. The results of this present study suggest that this diabetic MMP-tissue destruction cascade can be screened and detected in real-time and online by aMMP-8 PoC test at the dentist's office within 5 minutes. These patients with elevated aMMP-8 levels are associated with elevated HbA1c and thus may benefit from further referrals to their physicians in addition to their periodontal treatment. This would serve as a foundation for highly valuable communication between dental and medical clinicians, fostering enhanced collaboration and interprofessional cooperation. 40 Utilizing commercially available aMMP-8 PoC-testing is initially an extra expense but ultimately, it can become economical for patients and medical professionals. 42 If optimally utilized, aMMP-8 testing can generally improve oral health in a broad sense. This is achieved by the timely assessment of the risk for continuous destruction of the periodontal tissue (collagenolysis), and monitoring the disease activity of periodontitis patients to assess their increased need for periodontal disease prevention and treatment modalities (treatment prioritization), all of which slow down the progression of periodontitis and may aid in identifying developing periodontitis early enough.

The study had limitations, especially in terms of a smaller number of subjects in the more severe periodontitis category, hence the results need to be extrapolated with caution. Among the strengths of this study were the wide range of potential oral fluid biomarkers analyzed and compared with each other as well as the way the latest classification system of periodontitis, that is, the stage of periodontitis and grade of periodontitis was complemented with a variable of their combination, depicting simultaneously the severity and rate of progression (disease activity) of periodontitis. Stage and grade of periodontitis are the main attributes in the disease classification and as this study shows their combination may be a useful new variable to complement them in the future studies of biomarkers for periodontitis. More research is still required, for example, on the association between the grade of periodontitis and oral fluid biomarker levels in different stages of periodontitis with and without (pre)diabetes.

## Conclusion

Finally, the PoC/chairside diagnostic findings in this study are in agreement with previous findings and reveal that the tissue-destructive disease development in periodontitis and (pre)diabetes can be screened online and in real-time and diagnosed by a noninvasive aMMP-8 PoC diagnostic test.
