# Supplementary material for: Prediabetes Associates with Matrix Metalloproteinase-8 Activation and Contributes to the Rapid Destruction of Periodontal Tissues
Source: Eur J Dent. 2024 Oct 1;19(2):305–14. doi: 10.1055/s-0044-1788797 (PMC12020604; doi:10.1055/s-0044-1788797)
Supplement: Supplementary file 1 — Supplementary Material [file 10-1055-s-0044-1788797-s2443478.pdf]

Supplementary Table S1 Association of oral fluid biomarkers with periodontitis stage and periodontitis grade in 150 Greek adults

	No periodontitis ($N = 31$; median ± IQR)	Stage I ($N = 15$; median ± IQR)	Stage II ($N = 81$; median ± IQR)	Stage III ($N = 23$; median ± IQR)	*p*-Value (Stage)[a]	Pairwise comparisons (significant pairs; Stage)[b]	Grade A ($N = 14$; median ± IQR)	Grade B (GB) ($N = 91$; median ± IQR)	Grade C (GC) ($N = 14$; median ± IQR)	*p*-Value (Grade)[a]	Pairwise comparisons (significant pairs; Grade)[b]
aMMP-8 PoC test (ng/mL)	15.00 ± 0.00	15.00 ± 10.00	15.00 ± 13.45	39.34 ± 43.38	<0.001	NP–S2, NP–S3, S1–S2, S1–S3, S2–S3	15.00 ± 10.00	15.00 ± 18.90	28.33 ± 29.08	<0.001	GA–GB, GA–GC
aMMP-8 PoC test/NTP (ng/mL)	0.54 ± 0.06	0.58 ± 0.51	0.68 ± 0.69	1.66 ± 2.29	<0.001	NP–S2, NP–S3, S1–S3, S2–S3	0.59 ± 0.51	0.77 ± 0.70	1.30 ± 1.30	0.002	GA–GB, GA–GC
Total MMP-8 (ng/mL)	36.56 ± 76.29	22.87 ± 46.44	44.10 ± 80.04	50.96 ± 91.96	0.180	–	38.82 ± 37.49	39.36 ± 74.81	60.21 ± 140.45	0.499	–
PMN Elastase (ng/mL)	52.89 ± 195.34	166.59 ± 361.20	148.17 ± 308.55	249.32 ± 497.73	0.044	NP–S3	143.21 ± 224.27	175.13 ± 374.54	105.22 ± 314.20	0.521	–
MPO (μg/mL)	1.51 ± 2.49	1.24 ± 2.34	1.67 ± 2.84	2.64 ± 3.25	0.898	–	1.44 ± 2.99	1.68 ± 2.83	2.78 ± 3.40	0.901	–
MMP-9 (activation %)	20.00 ± 40.00	40.00 ± 20.00	40.00 ± 40.00	40.00 ± 40.00	0.271	–	40.00 ± 20.00	40.00 ± 20.00	60.00 ± 50.00	0.013	GB–GC
Total MMP-9 (ng/mL)	83.53 ± 104.01	58.72 ± 63.72	68.29 ± 130.14	85.17 ± 128.04	0.791	–	79.67 ± 103.26	67.55 ± 128.11	88.45 ± 75.71	0.706	–
TIMP-1 (ng/mL)	199.21 ± 151.42	207.37 ± 174.94	145.72 ± 136.88	150.57 ± 141.14	0.074	–	190.13 ± 190.41	147.69 ± 137.95	183.51 ± 121.83	0.148	–
IL-6 (pg/mL)	0.00 ± 2.00	2.76 ± 15.92	2.28 ± 8.02	1.34 ± 5.06	0.084	–	0.00 ± 10.18	2.28 ± 8.48	2.76 ± 5.20	0.658	–
Calprotectin (μg/mL)	14.76 ± 16.32	13.23 ± 25.50	13.98 ± 18.31	19.76 ± 26.75	0.413	–	15.02 ± 37.28	14.29 ± 20.45	14.57 ± 36.52	0.761	–
aMMP-8/TIMP-1 molar ratio	1.32 ± 1.34	1.23 ± 1.66	1.92 ± 1.75	2.02 ± 2.91	0.037	–	1.79 ± 1.70	1.89 ± 1.84	1.89 ± 1.10	0.642	–
tMMP-8/TIMP-1 molar ratio	0.07 ± 0.12	0.05 ± 0.12	0.08 ± 0.25	0.17 ± 0.40	0.012	S1–S3	0.07 ± 0.19	0.08 ± 0.26	0.16 ± 0.28	0.564	–
tMMP-9/TIMP-1 molar ratio	0.13 ± 0.22	0.11 ± 0.16	0.12 ± 0.25	0.13 ± 0.31	0.551	–	0.13 ± 0.21	0.12 ± 0.23	0.13 ± 0.34	0.642	–

Abbreviations: aMMP-8, active-matrix metalloproteinase-8; GA, grade A; GB, grade B; GC, grade C; IQR, interquartile range; MMP, matrix metalloproteinase; NP, no periodontitis; /NTP, per number of teeth present; PoC, point-of-care; S1, stage 1; S2, stage 2; S3, stage 3.
[a]Kruskal–Wallis test.
[b]Dunn–Bonferroni test.

 © 2024. The Author(s).
